# Genetic co-expression networks contribute to creating predictive model and exploring novel biomarkers for the prognosis of breast cancer

**DOI:** 10.1038/s41598-021-84995-z

**Published:** 2021-03-31

**Authors:** Yuan-Kuei Li, Huan-Ming Hsu, Meng-Chiung Lin, Chi-Wen Chang, Chi-Ming Chu, Yu-Jia Chang, Jyh-Cherng Yu, Chien-Ting Chen, Chen-En Jian, Chien-An Sun, Kang-Hua Chen, Ming-Hao Kuo, Chia-Shiang Cheng, Ya-Ting Chang, Yi-Syuan Wu, Hao-Yi Wu, Ya-Ting Yang, Chen Lin, Hung-Che Lin, Je-Ming Hu, Yu-Tien Chang

**Affiliations:** 1 Division of Colorectal Surgery, Department of Surgery, Taoyuan Armed Forces General Hospital, Taoyuan, Taiwan; 2 Department of Biomedical Sciences and Engineering, National Central University, Taoyuan, Taiwan; 3 Division of General Surgery, Department of Surgery, Tri-Service General Hospital, National Defense Medical Center, Taipei, Taiwan; 4 Department of Surgery, Songshan Branch of Tri-Service General Hospital, National Defense Medical Center, Taipei, Taiwan; 5 Department of Otolaryngology-Head and Neck Surgery, Tri-Service General Hospital, National Defense Medical Center, Taipei, 11490 Taiwan; 6 Division of Gastroenterology, Department of Medicine, Taichung Armed Forces General Hospital, Taichung, Taiwan; 7 School of Nursing, College of Medicine, Chang Gung University, Taoyuan, Taiwan; 8 Department of Pediatrics, Chang Gung Memorial Hospital, Taoyuan, Taiwan; 9 Department of Nursing, Chang Gung Memorial Hospital, Tao-Yuan, Taiwan; 10 Division of Medical Informatics, Department of Epidemiology, School of Public Health, National Defense Medical Center, Taipei, Taiwan; 11 Big Data Research Center, College of Medicine, Fu-Jen Catholic University, New Taipei City, Taiwan; 12 Department of Public Health, College of Medicine, Fu-Jen Catholic University, New Taipei City, Taiwan; 13 Department of Public Health, China Medical University, Taichung City, Taiwan; 14 Department of Healthcare Administration and Medical Informatics College of Health Sciences, Kaohsiung Medical University, Kaohsiung, Taiwan; 15 Graduate Institute of Clinical Medicine, College of Medicine, Taipei Medical University, Taipei, Taiwan; 16 Cell Physiology and Molecular Image Research Center, Wan Fang Hospital, Taipei Medical University, Taipei, Taiwan; 17 Graduate Institute of Medical Sciences, National Defense Medical Center, Taipei, Taiwan; 18 Graduate Institute of Life Sciences, National Defense Medical Center, Taipei, Taiwan; 19 Center for Biotechnology and Biomedical Engineering, National Central University, Taoyuan, Taiwan; 20 Hualien Armed Forces General Hospital, Xincheng, Hualien, 97144 Taiwan; 21 Division of Colorectal Surgery, Department of Surgery, Tri-Service General Hospital, National Defense Medical Center, Taipei City, Taiwan; 22 School of Medicine, National Defense Medical Center, Taipei City, Taiwan

**Keywords:** Cancer genetics, Breast cancer

## Abstract

Genetic co-expression network (GCN) analysis augments the understanding of breast cancer (BC). We aimed to propose GCN-based modeling for BC relapse-free survival (RFS) prediction and to discover novel biomarkers. We used GCN and Cox proportional hazard regression to create various prediction models using mRNA microarray of 920 tumors and conduct external validation using independent data of 1056 tumors. GCNs of 34 identified candidate genes were plotted in various sizes. Compared to the reference model, the genetic predictors selected from bigger GCNs composed better prediction models. The prediction accuracy and AUC of 3 ~ 15-year RFS are 71.0–81.4% and 74.6–78% respectively (rfm, ACC 63.2–65.5%, AUC 61.9–74.9%). The hazard ratios of risk scores of developing relapse ranged from 1.89 ~ 3.32 (p < 10^–8^) over all models under the control of the node status. External validation showed the consistent finding. We found top 12 co-expressed genes are relative new or novel biomarkers that have not been explored in BC prognosis or other cancers until this decade. GCN-based modeling creates better prediction models and facilitates novel genes exploration on BC prognosis.

## Introduction

Breast cancer (BC) is a major health threat to women worldwide and supposedly results from stochastic molecular changes over long periods^1^. Resistance to therapy is not only common^2,3^ but expected as the progression of BC occurs^4,5^. Understanding the underlying molecular mechanisms^2,6^ and identifying novel genome profiles will aid in the development of therapies^7,8^. Microarray analyses of gene profiles offer potential prognostic information and identify differentially expressed genes (DEGs) for the prognosis of newly diagnosed BC^9–14^. However, since BC is a disease of complex coordinated molecular activities, it should be determined by the coordination of GCNs rather than DEGs. The information behind the GCN is of great importance^15^. GCN analyses are proven to be an efficient and systemic method to discuss biological network mechanisms or for the identification of novel biomarkers of BC^10,11,16–22^. In addition, when accompanied by the integration of publicly available genomic studies, they provide more accurate and robust results^16,23^. Therefore, we proposed GCN-based modeling to create better prediction models or gene panels of BC prognosis and explore novel biomarkers and putative functional pathways.

## Results

### Descriptive statistics of BC patients of validation data sets

There are 394 recurrence patients and 662 no recurrence patients and the average follow-up time are 8.86 ± 3.19 and 3.23 ± 2.85 years respectively. Except for ER status, younger age (OR 0.98 p < 0.007), bigger tumor size (OR 1.27 p < 0.001), positive lymph node (OR 1.27 p < 0.001) and higher grades (OR 2.7 ~ 2.9 p < 0.001) are statistically associated with BC relapse using univariable logistic regression (Table 1).

**Table 1:** The clinical characteristics of BC patients of validation data sets.

	RFS_event				OR	p
	No (n = 662)		Yes (n = 394)			
	Mean	sd	Mean	sd		
Follow-up time (year)	8.86	3.19	3.23	2.85	0.559	< 0.001
Age	59	13	56	13	0.984	0.007
Tumor size	2.17	1.13	2.46	1.08	1.267	0.001
	n	%				
**ER**						
Negative	160	20.6%	97	21.9%	0.925	0.591
Positive	617	79.4%	346	78.1%		
**Lymph node**						
Negative	629	87.0%	340	78.5%	1.830	
Positive	94	13.0%	93	21.5%		< 0.001
**Grades**						
1	133	26.0%	36	11.1%		
2	221	43.2%	173	53.2%	2.892	< 0.001
3	158	30.9%	116	35.7%	2.712	< 0.001

*RFS* relapse-free survival, *ER* estrogen receptor, *sd* standard deviation. Odds ratio (OR) and p values are analyzed using univariable logistic regression.

### GCN-based models outperform the reference model

In the validation data sets, there are only five clinical-pathological characteristics of ER status, lymph node, grade and tumor size available. There are many missing values in these variables. Lymph node status has adequate data and is an important factor to BC recurrence^24–26^. Therefore, only lymph node was included while modeling. Models were trained using stepwise cox hazard proportional regression. Model 1 served as a reference model for comparison, and its input predictors were 34 key candidate genes (KCGs). The input predictors of Model 2–4 were genes in GCNs with various criteria of r values of 0.82, 0.80 and 0.79. The final optimal models of Models 1–4 comprised 6, 13, 17, and 34 significant genes (p < 0.05) (Table 2). Model 5, created by the stepwise network modeling (SNM), contained eight genes. While the GCNs became larger, more important genes were included in the GCN-based models. The total R squared values increased from 0.05 to 0.21 in order from Models 1–4 and showed a significant goodness of fit (Likelihood ratio test p = 0 ~ 0.001) (Table 3). In addition, we carried out research on adopting other clinical variables and statistical method. In the entry model of multivariable logistic regression of five clinical-pathological factors (Supplementary Table 1), tumor size is the only significant factor related to BC recurrence that we included as a mandatory variable during modeling. The AUC and AUC are increasing from Model 1 to Model 4 in Supplementary Table 2 in line with above findings. In addition, we found that models included clinical variables had less prediction power than those without it.

**Table 2:** The number of genes in the GCNs.

r	Recurrence	No recurrence	Total number of unique genes
Ref	34	34	34
> 0.82	79	131	137
> 0.80	110	216	221
> 0.79	133	310	443

**Table 3:** Characteristics of GCN-based models.

	Model 1	Model 2	Model 3	Model 4	Model 5^a^
Total # of genetic factors	34	137	221	443	46
# of genes in the model	6	13	17	34	8
ACC	63.2–65.5	66.5–70.3	66.6–74.4	78.2–82.5	66.5–74.4
AUC	61.9–64.2	64.2–75	65.2–75.1	68.7–77.1	64.1–74.8
R square	0.05	0.09	0.12	0.21	0.08
goodness of fit	< 0.001	1.21E−11	2.22E−16	~ 0	2.77E−12

*ACC* accuracy, *AUC* area under the curve.

^a^Model created using the approach of SNM.

**Figure 1:**
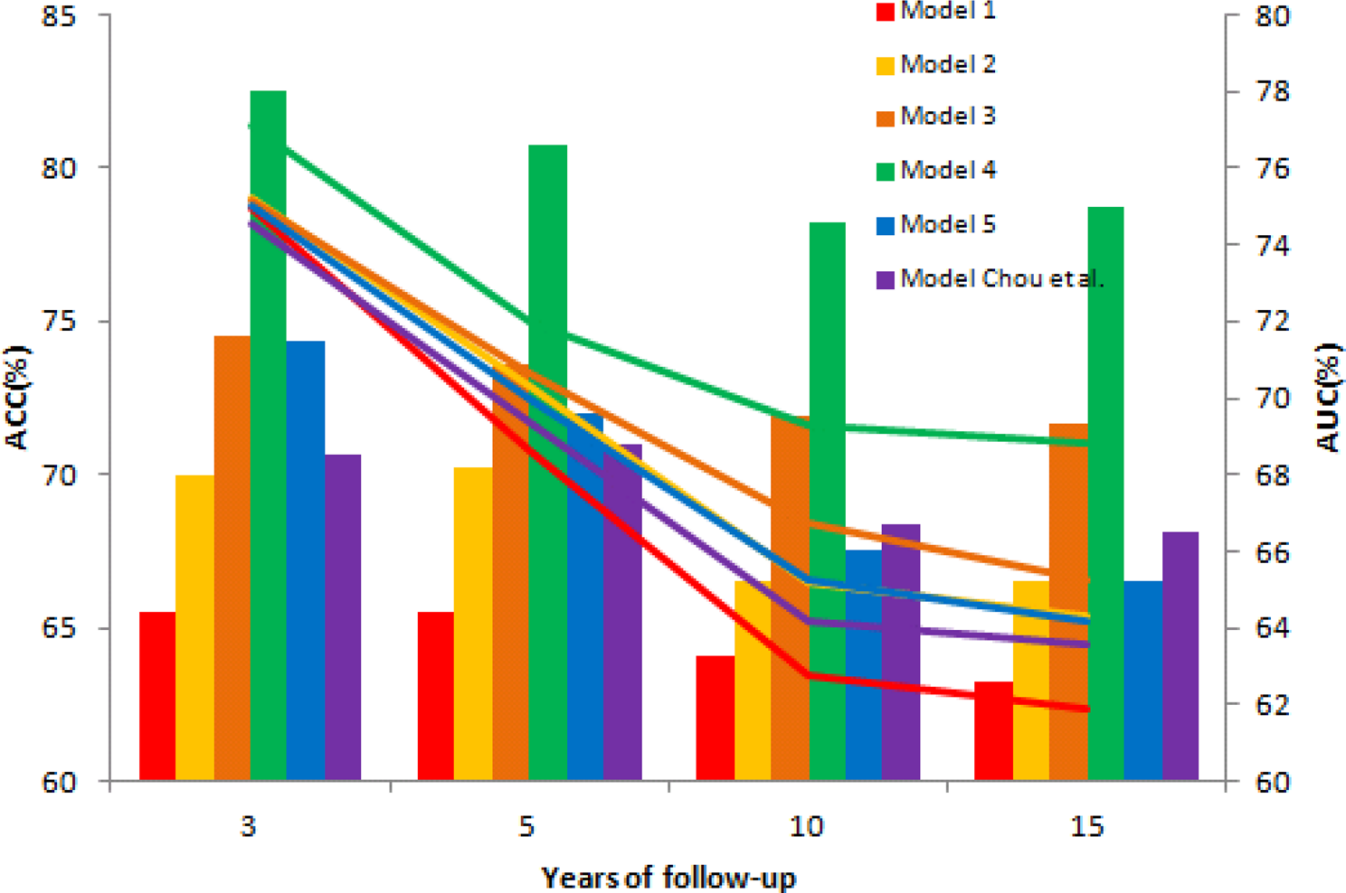
The ACCs (bar chart) and AUCs (histogram) of Model 1–5 and Model from Chou’s study^28^ for predicting the 3, 5, 10 and 15-year RFS in BC.

We evaluated the prediction performance of the models on 3, 5, 10 and 15 years of BC RFS using a logistic regression and ROC curve analysis. The results showed that Model 2–4 outperformed Model 1 (Accuracy; ACC 63.2–65.5%, Area under the Curve of ROC; AUC 61.9–64.2) measured by time-dependent accuracy (Supplementary Fig. 1) and time-dependent AUC (Supplementary Fig. 2). Additionally, the top two precise models were Model 4 (ACC 78.2–82.5%; AUC 68.7–77.1%) and Model 3 (ACC 66.6–74.4%, AUC 65.2–75.1%) (Fig. 1). All the models reached the best prediction on the 3-year RFS.

### The risk scores of GCN-based models succeed in predicting RFS of BC

**Figure 2:**
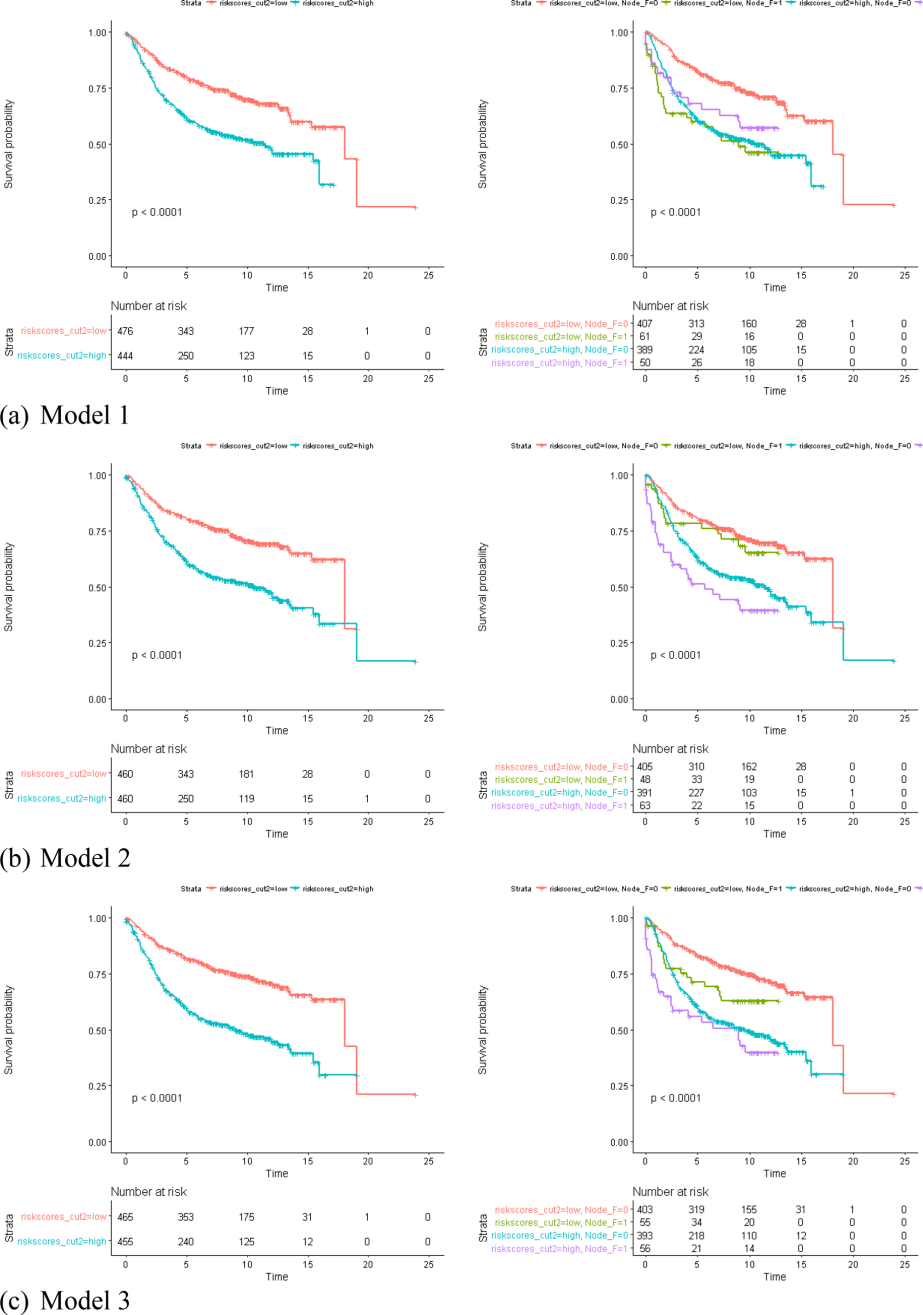

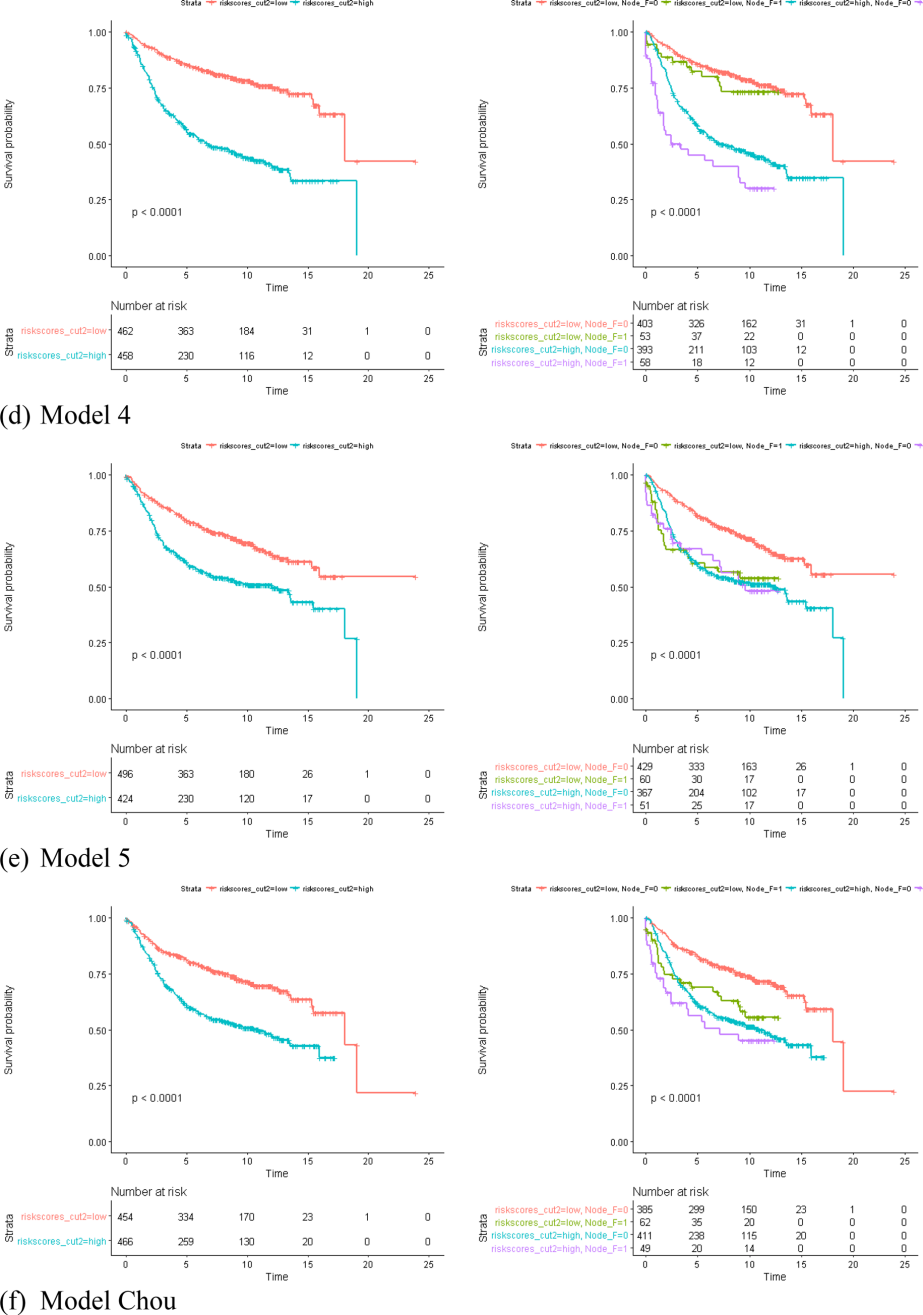
Partial cox regression plots of Models 1–5 and Model from Chou’s study^28^.

**Figure 3:**
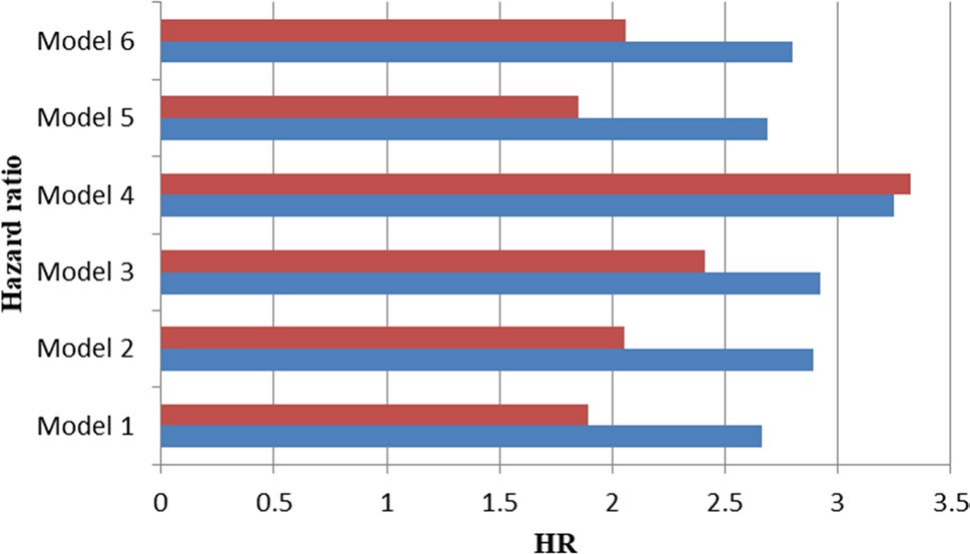
Cox regression of the risk scores of each model on predicting the risk of relapse for breast cancer patients. Red bars are the hazard ratio (HR) of categorical risk scores of each model; blue bars are the hazard ratio (HR) of continuous risk scores of each model.

We used a partial Cox hazard proportional regression^27^ to compute the risk score of each model. The risk scores were used to predict the RFS of BC in the form of continuous and categorical variables (Fig. 2). All the risk scores significantly predicted the RFS of BC, and HRs ranged from 1.89 ~ 3.32 (p < 10^–8^) under the control of the node status. The risk score of Model 4 has the best discrimination for whether the high/low risk group developed recurrence (high risk group: HR 3.25, p ~ 0) under the control of the node status (Fig. 3 and Supplementary Table 3). According to the time-dependent the prediction error and AUCs in Supplementary Figs. 1, 2, model 4 was also the most precise prediction model followed by Model 3, Model 2, Model 5/Model 6, and Model 1, in that order. In summary, the GCN-based models outperformed the reference model, and the larger GCN-based model performed better.

### Validation of model prediction using independent data sets

We used the public independent mRNA microarray data of 1056 eligible primary BC tissues from KM plotter websites to do external validation. The outcomes are concordant with the modeling results. Model 4 has the best AUC 72.1–74.8% on predicting the RFS in 3, 5 and 10 years (Table 4 and Supplementary Fig. 3). All models predict best on 3-year RFS.

**Table 4:** The AUC of model validation using independent data sets.

n-year RFS	Model 1	Model 2	Model 3	Model 4	Model 5
3	69.2 [65.3;73.1]	69.6 [65.6:73.6]	70.6 [66.6;74.6]	74.8 [71.1;78.6]	69.7 [65.8;73.6]
5	68 [64.5;71.6]	68.7 [65.0;72.3]	69.5 [65.9;73.1]	74.6 [71.3;78.0]	68.1 [64.5;71.6]
10	66.8 [62.7;70.9]	67.0 [62.9;71.1]	67.4 [63.4;71.5]	72.1 [68.2;75.9]	66.5 [62.3;70.6]

Models are under the control of node status.

### GCN-based models outperform with less genetic predictors

We integrated the 46 significant genes from Model 1 to 4 and filtered out the most important genes using the criteria of uni-variable cox hazard proportional regression p < 0.001, HR > 1.5 or HR < 0.7 (= 1/1.5) under the control of the node status. Finally, we chose 12 genes and used stepwise forward cox hazard proportional regression to create Model 5, which was composed of eight genes, including *AACS, C10orf5, CCNE2, EEF1E1, IDUA, LMNB1, MGC27165,* and *RORC*. In comparison to the 21-gene model (Model 6) from Chou’s study^28^ using the same integrated GSE data sets, the GCN-based Model 5 (ACC 66.5–74.4%, AUC 64.1–74.8%) predicted as accurately as Model 6 (ACC 68.1–70.9%, AUC 63.4–74.5%) with only eight genetic predictors and had a better AUC (Fig. 1). This suggested that the GCN-based models not only reduced the dimension of the predictors but also filtered out the most representative and important genetic predictors.

### Larger GCNs effectively provide more information on the novel genes related to recurrence

We also confirmed that larger GCNs effectively provided more information on the novel genes related to recurrence. In Models 2–4, we found some highly co-expressed with 34 candidate genes that statistically associated with recurrence, including Model 2—*CCNA2*, Model 3—*CCNA2*, *IDUA, MGC27165, CCNE2, KIF14* and *C10orf56* and Model 4—*IDUA, MGC27165, CCNE2, KIF14, EBP* and *RORC* (Supplementary Table 4).

### Predictive pathway of importance novel genes

**Figure 4:**
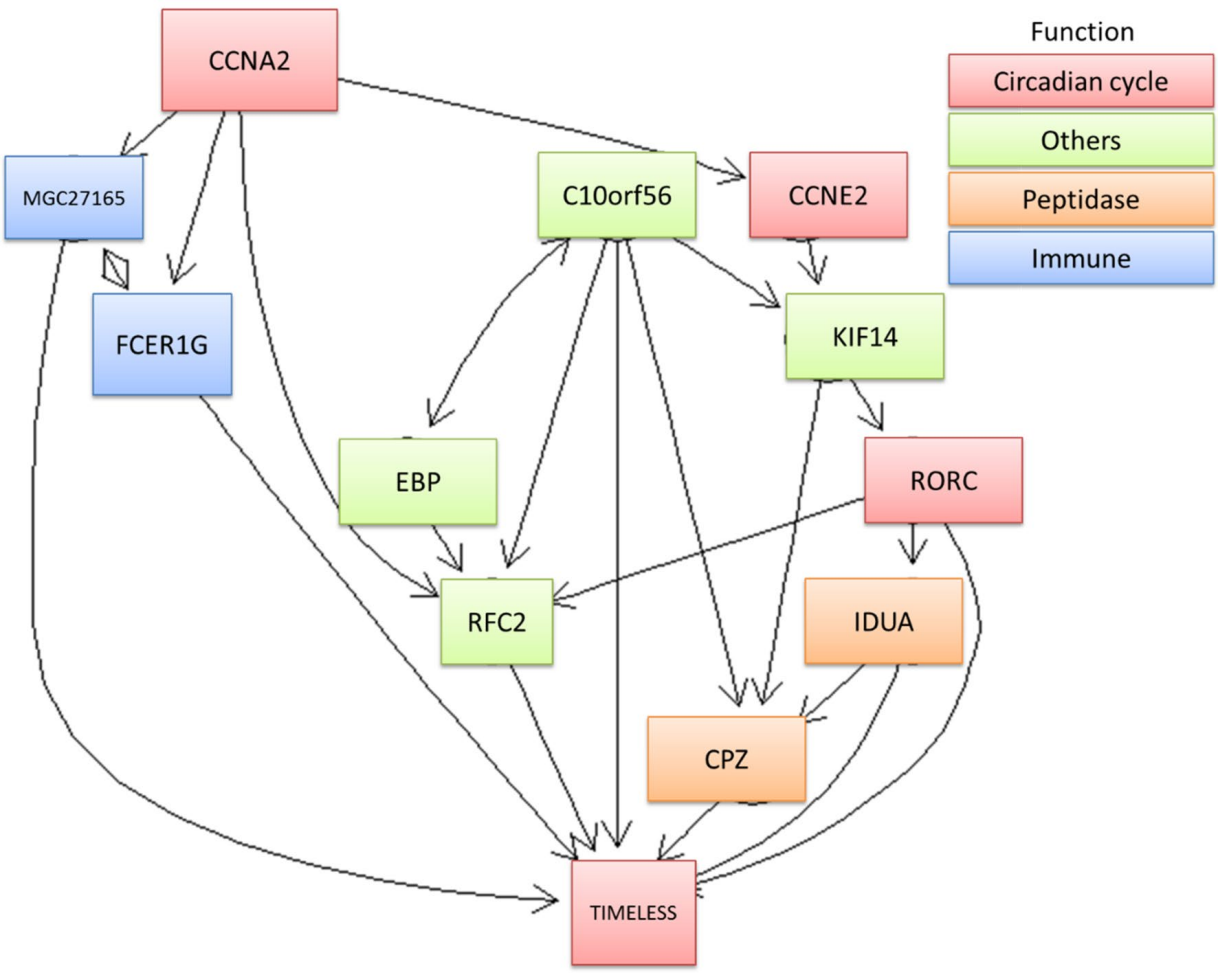
The predictive pathways of top 12 important co-expressed genes.

The importance of the genes related to RFS was assessed by computing the proportion of the chi-square of each gene in each model. We plotted the relative importance of the genes in the order of the sum and average of importance indices (Supplementary Table 5 and Supplementary Fig. 4). Top 12 important genes are selected to plot the predictive pathways (Fig. 4). The function of these genes are clustered into 4 groups "Circadian Cycle (*CCNA2, CCNE2, RORC, TIMELESS*)", "Peptidase (*IDUA, CPZ*)", "Immune (*MGC27165, FCER1G*)" and others (*C10orf56, KIF14, EBP, RFC2*). The dysregulation of *CCNA2* triggers the consequent reaction of other genes and leads to *TIMELESS* that influence the recurrence of BC.

## Discussion

Gene expression profiling of BC has shifted from differentially expressed genes (DEGs) to GCN analyses. GCN analyzes have been shown to aid comprehensive understanding of genomes regulation^10,16,17^. In this study, we proposed GCN-based modeling and SNM to create better prediction models of RFS in BC and explored novel significant co-expression genes. This is the first study to evaluate the prediction of GCN-based models created by various sizes of GCNs. We found that GCN-based modeling from larger GCNs created good prediction gene panels for BC recurrence either in the training and validation datasets (Table 4 and Fig. 3). Genetic predictors selected merely from the DEGs (DEG-based model) may miss key genetic information. GCN-based models can make up for the weakness and increase the prediction accuracy.

Though SNM model (Model 5) did not reach the best prediction outcome yet it predicted as accurately as Model 6 (from Chou’s study^28^), which created by our previous study contain 21 genes with less genetic predictors (eight genes). It indicated that GCN-based and SNM modelings are better approaches than DEG-based model (Model 1) for creating good gene panels with fewer genes but higher prediction accuracy.

Analysis of integrated microarray data sets facilitates the gene expression profiling of BC, but the lack of complete clinical characteristics is a big issue. Besides, most of the GCN studies^16,18,29–31^ are analyzed on already known biological pathways, the gene–gene interaction from text mining science articles or re-calculation using public genome data. However, those studies sometimes miss the information of unknown GCNs or biomarkers whose function has not yet been identified.

Our GCN-based modeling can make up for these deficiencies. 34 KCGs were selected by choosing the overlapping genes in five BC prognosis-related studies^28,32–36^. It is suggested that these genes play essential and stable roles in the mechanism of recurrence of BCs. The GCNs of 34 KCGs were assumed to include more significant novel genes related to recurrence even without considering the clinical characteristics. Therefore, even though we only considered one clinical variable node status, the results are still informative.

In general, the values of the time-dependent AUCs, time-dependent ACCs and HRs increased in the order of Model 4, Model3, Model 2, and Model 1. Model 4 predicted the RFS most accurately at any time point of 3, 5, 10 and 15 years. All the models applied in the short-term 3-year RFS performed with the best prediction (Fig. 1). GCN-based models effectively provide more novel and significant genetic information related to BC recurrence while the GCNs grow larger. The top 12 important genes from all the models were identified to plot the predictive pathway (Fig. 4) which started from *CCNA2* and finished on *TIMELESS*. Functional annotations of 12 important genes are: (1) circadian cycle (*CCNA2*, *CCNE2*, *RORC*, *TIMELESS*); (2) immune (*MGC27165, FCER1G*); (3) peptidase [*IDUA, CPZ*]; (4) kinesin (*KIF14*); (5) DNA repair (*RFC2*); (6) membrane binding protein (*EBP*); and (7) nucleic acid binding and poly(A) RNA binding (*C10orf56*)^37^. Through literature review, these genes are biologically related to BC or other cancers^11,38–56^ in line with our bioinformatics findings.

It is notable that the role of *TIMELSS* in the progression of BC has not been well-characterized until these few years^57–60^. The overexpression of *TIMELSS* upregulated the expression and the trans-activity of the well-known oncogene *MYC*. Inhibition of *MYC* significantly blocked the effects of *TIM* on cancer stem cell population, cell invasion and anchor-independent cell growth^60^. Therefore, the functional gene–gene interactions in the pathway warrant further study to understand more about the mechanism of BC progression.

*CCNA2* is a regulator of the cell cycle^61^. Its overexpression increased BC proliferation^62^ and is an early/transient/proliferation response biomarker^63^ for the prognosis of ER + BC and the monitoring of tamoxifen efficacy^38^.

*CCNE2* has the same function as *CCNA2*. It might play an important role in acquired trastuzumab resistance in HER2 + breast cancer ^40^.

*RORC (RAR Related Orphan Receptor C)* is a suppressor gene^46,64,65^ associated with BC carcinogenesis. An RORC agonist suppresses breast cancer cell viability, migration, the EMT transition (microsphere outgrowth) and mammosphere-growth^64^.

*IDUA(Iduronidase, Alpha-L) *is associated with visceral organ metastatic disease in breast cancer^49^.

*CPZ(Carboxypeptidase Z) *expression was significantly lower ovarian cancers and may be relevant to the biology of high-grade serous ovarian cancers^66^. But no study discussed its role in BC.

*MGC27165(IGHA1) *was associated with BC survival time^47^ and was suppressed in triple negative breast cancer patients with poor prognosis^11^.

*FCER1G* (High-Affinity Immunoglobulin Epsilon Receptor Subunit Gamma)

Receptors for immunoglobulins [Fc-receptors (FcRs)] are widely expressed throughout the immune system^67^ and are related to antibody-based therapeutics, such as trastuzumab^68^.

*C10orf56 (Chromosome 10 open reading frame 56, ZCCHC24) *participated in tumorigenesis by inhibiting BET family proteins^69^. Its specific methylation pattern affected the expression level and was related to BC subtypes detection^56^.

*KIF14 (Kinesin Family Member 14)* is a prognostic predictor of BC ^53,54,70^. It promotes AKT phosphorylation and contributes to chemoresistance in triple-negative breast cancers^51,52^.

*RFC2 (Replication Factor C Subunit 2)* is only mentioned in one study for its indirect association with BC and involvement in DNA repair ^55^.

*EBP* (emopamil-binding protein) is a human sterol isomerase (hSI) that is associated with a poorer BC disease-free survival^71^. SR31747A (sigma receptor ligand) binds with *EBP* and other proteins to exhibit antitumoral activity^72^. Few studies discuss its role in BC.

There are many missing data of clinical variables in validation data sets obtained from public database. Only significant lymph node status which had sufficient samples was included while modeling. It’s the limitation of the study. However, it will not alter the main findings that bigger genetic co-expression networks are more likely to produce good prediction models. In addition, all the significant identified biomarkers were reported to be associated with BC or other cancers. However, it will be more comprehensive to include as many clinical factors as possible for analysis.

## Conclusion

We proposed GCN-based modeling and SNM method to construct more precise models than DEGs-based models with fewer genetic predictors. The results showed that all the GCN-based models outperformed the DEG-based model. Our framework systematically facilitates the discovery of novel co-expressed genes for BC prognosis without prior biological information of genes. We succeeded in finding relatively new co-expressed genes, such as *TIMELESS, IDUA, CPZ, MGC27165, C10orf56* and so on that were found to be associated with BC or other cancers until this decade. In general, our GCN-based models and SNM facilitate studies to create prediction models and discover novel biomarkers.

## Methods and materials

### Microarray data sets

**Figure 5:**
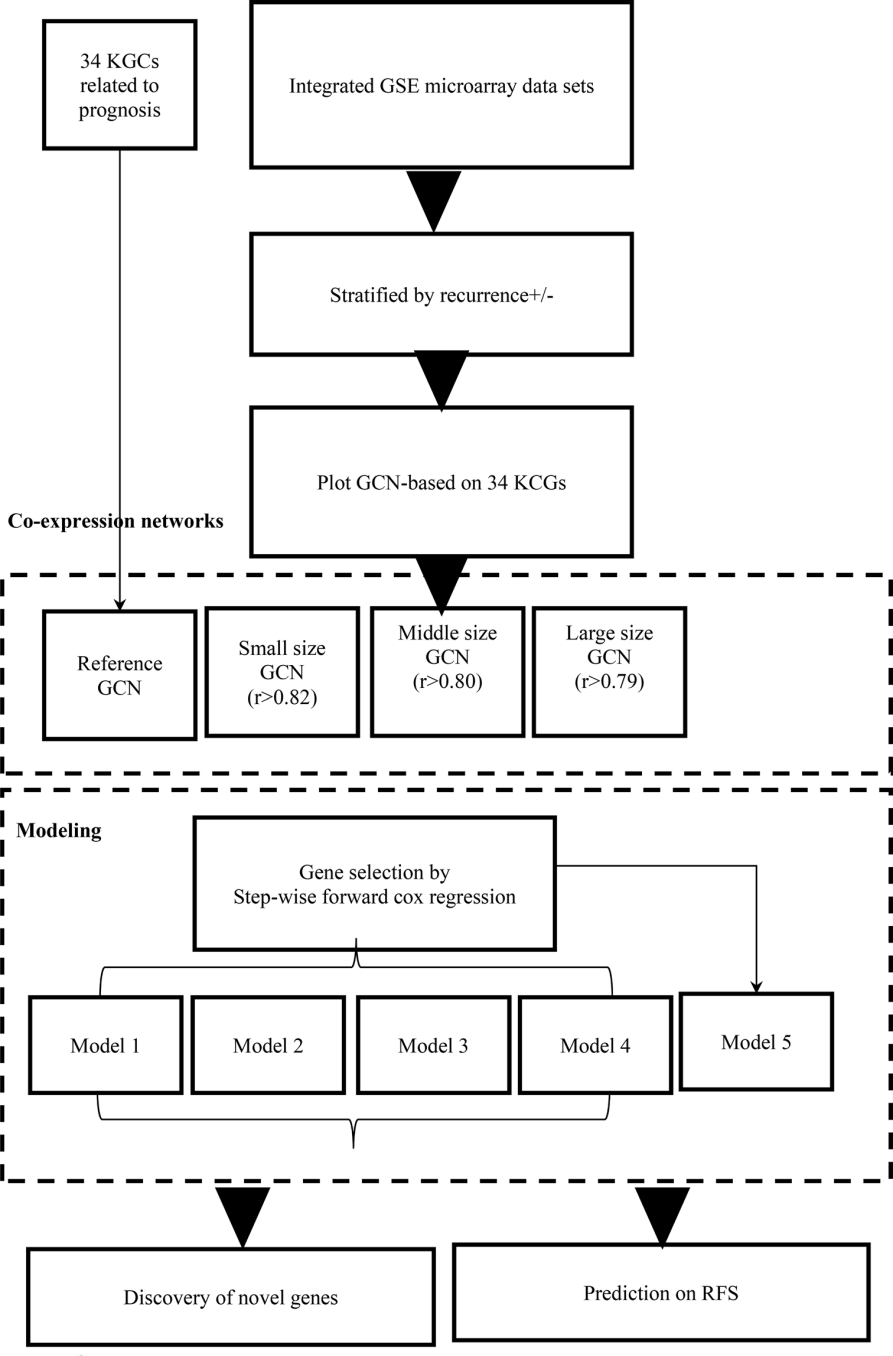
Workflow of the study.

The mRNA microarray data of BC retrieved from Chou’ study^28^, including GSE 2034 (n = 286)^32^, GSE 2990 (n = 189)^34^, GSE 4922 (n = 249)^35^, and GSE 7390 (n = 198)^33^ of the NCBI GEO, and comprised a total of 922 cases and 13,452 genes, with 354 cases showing recurrence of breast cancer (38%) at the end of follow-up. A total of 111 node-positive cases (12%) and a total of 796 negative cases (86%) were included. The four data sets showed no difference in determining the distribution of recurrence (Supplementary Table 6). The pre-processing of the microarray data was denoted in Chou’s study^28^. They used quantile normalization^73,74^ to normalize all the mRNA expression values and calculated the median probe expression in a gene to represent the mRNA expression level. GSE7390^33^ was used as the reference standard, and the other three data sets were log transformed to fit the former distribution. The workflow of study is shown in Fig. 5.

### Validation data sets

mRNA validation data sets were derived from KM plotter website (https://kmplot.com/) comprising 1809 BC tumor samples and 13,747 genetic characteristics. 1056 samples were included after excluding missing data (recurrence n = 394, no recurrence n = 662).

### Thirty-four candidate genes

We chose the significant gene signatures from five studies (Supplementary Table 7). A comparison of the top 100 significant genes (Supplementary Table 8) related to BC recurrence from our previous study^28^ revealed 34 identical genes. (Supplementary Table 8) These 34 candidate genes have a stable and dynamic influence on the occurrence of recurrence of BC, and thus, these candidates were used to establish various GCNs.

### Plot GCNs

We used R version 3.2.2 software (http://www.r-project.org)^75^ for the statistical computing and graphics. The GCN was developed using the package visNetwork^76^. The correlation coefficient, hierarchical clustering, coefficient of variation, and Cox hazard proportional regression were computed using the *cor, hclust, co.var*, and *coxph* functions. Due to variations in genotype and recurrence, the data were divided into two data sets by recurrence status for analysis.

The 34 KCGs were essential nodes in the GCNs. A Spearman's rank correlation analysis was applied on the 34 KCGs and all the other genomes of 13,418 genes. The genes with an r value over the threshold we set were selected and drawn in the GCNs. If the edges, starting from each node (gene) in the GCNs, were over two, only the most associative two were kept in order to identify the most important GCNs.

The regulation of the genetic networks of recurrence versus no recurrence is different. The GCNs for recurrence and no recurrence cases were drawn separately. The regulation of the recurrence of BCs was what we cared most about. Therefore, we plotted 2–4 times the size of 34 KCG GCN of recurrence data with the correlation thresholds of 0.82, 0.80 and 0.79 (Table 2).

### Create GCN-based cox hazard proportional regression models and SNM

We obtained four groups of genetic predictors of four GCNs under r thresholds of 0.82, 0.80 and 0.79 to create prediction models of RFS in BCs (Table 2). Stepwise forward cox hazard proportional regression was used to select significant genes which variance inflation factor (VIF) < 10, a chosen significance level for entry (SLE) = 0.08 and the chosen significance level for stay (SLS) = 0.05. We gathered all significant genes from Models 1–4 and filtered out the most important genes based on the criteria of the uni-variable cox hazard proportional regression p < 0.001, HR > 1.5 or HR < 0.7 (1/1.5) under the control of the node status. Using these important genes, We these important genes and stepwise forward cox hazard proportional regression to create model 5. We named the procedure used to create Model 5 as stepwise network modeling (SNM).

### Partial cox hazard proportional regression

To compare the prediction of the various models, we used a partial cox hazard proportional regression for constructing the mutually uncorrelated components of the genetic predictors in each model using the function of PCRf developed by Li and Gui^27^. This method was useful in building a parsimonious predictive model that accurately predicted the survival based on the mRNA expression profile. Predictive components whose p values were less than 0.05 (uni-variable Cox hazard proportional regression) were selected to rebuild a model [as shown in formula (1)]. The risk scores were computed by summing up the multiplication of the coefficient and selecting the component scale in the Cox hazard proportional regression [as shown in formula (2)]. Since the mean of each component scale was zero, we set zero as the cut-off point to categorize the patients into the high/low risk score groups. The Cox hazard proportional regression of the components is written as shown in formula (1). xi is the component extracted by the partial Cox hazard proportional regression. The risk scores were computed by formula (2).

1 $${\text{Hazard }}\,{\text{ratio}}\left( {\text{t}} \right) = {\text{ exp}}\left( {\alpha + \beta_{{1}} \chi_{{{\text{i1}}}} + \beta_{{2}} \chi_{{{\text{ik}}}} + \cdots + \beta_{{\text{k}}} \chi_{{{\text{ik}}}} } \right),$$

2 $${\text{riskscore}} = \beta_{{1}} \chi_{{{\text{i1}}}} + \beta_{{2}} \chi_{{{\text{ik}}}} + \cdots + \beta_{{\text{k}}} \chi_{{{\text{ik}}}} .$$

### Time-dependent AUC and prediction error

The area under the curve (AUC) of the ROC curve is a well-established indicator for assessing how well a prediction model performs. AUC ranges from 0 to 1. A model whose predictions are 100% wrong has an AUC of 0; one whose predictions are 100% correct has an AUC of 1^77^. The classical approach of the AUC analysis considers the event (disease) status and marker value to be fixed over time. However, in practice, both the disease status and marker value change over time. Thus, a time-dependent AUC is more appropriate^78^. We evaluated the prediction performance of each model by computing the time-dependent AUC and prediction error using the R package of "survAUC"^79^.

### Predictive pathway graph

Understanding cause-effect relationships between variables is of primary interest in cancer science. Usually, experimental intervention is used to confirm these relationships, but this can be infeasible because of time and cost. However, Kalisch et al.^80^ introduced "*pcalg*" to effectively explore causal relationships of important biomarkers in BC recurrence. Therefore, we used the mRNA dataset and R packages of "*pcalg*" and "*Rgraphviz*" to plot the predictive pathway. The alpha was set at 0.01.

### Ethics approval and consent to participate

The data used in the article are public available data from NCBI GEO (https://www.ncbi.nlm.nih.gov/geo/) and KM plotter (https://kmplot.com/analysis/). According to item two of the regulation "得免取得研究對象同意之人體研究案件範圍(Scope of human research cases exempt from obtaining consent) " (https://www.mohw.gov.tw/dl-45112-b708e126-a9c4-4842-ac83-ba6561948a2f.html), it denotes that " Use legally publicly known information and use the information for its publicly known purpose " meets the scope of the exemption.

### Consent for publication

None.

## Supplementary Information

Supplementary Information.

## Data Availability

The data sets used during the present study are available from the corresponding author on reasonable request and can be downloaded in GEO Data sets (https://www.ncbi.nlm.nih.gov/gds/).

## Abbreviations

GCN: Genetic co-expression network
SNW: Stepwise network modeling
DEGs: Differentially expressed genes
DAG: Directed acyclic graph
KCG: Key candidate genes
