# Publisher Correction: Genetic co-expression networks contribute to creating predictive model and exploring novel biomarkers for the prognosis of breast cancer

**DOI:** 10.1038/s41598-021-89147-x

**Published:** 2021-05-04

**Authors:** Yuan-Kuei Li, Huan-Ming Hsu, Meng-Chiung Lin, Chi-Wen Chang, Chi-Ming Chu, Yu-Jia Chang, Jyh-Cherng Yu, Chien-Ting Chen, Chen-En Jian, Chien-An Sun, Kang-Hua Chen, Ming-Hao Kuo, Chia-Shiang Cheng, Ya-Ting Chang, Yi-Syuan Wu, Hao-Yi Wu, Ya-Ting Yang, Chen Lin, Hung-Che Lin, Je-Ming Hu, Yu-Tien Chang

**Affiliations:** 1 Division of Colorectal Surgery, Department of Surgery, Taoyuan Armed Forces General Hospital, Taoyuan, Taiwan; 2 Department of Biomedical Sciences and Engineering, National Central University, Taoyuan, Taiwan; 3 Division of General Surgery, Department of Surgery, Tri-Service General Hospital, National Defense Medical Center, Taipei, Taiwan; 4 Department of Surgery, Songshan Branch of Tri-Service General Hospital, National Defense Medical Center, Taipei, Taiwan; 5 Department of Otolaryngology-Head and Neck Surgery, Tri-Service General Hospital, National Defense Medical Center, Taipei, 11490 Taiwan; 6 Division of Gastroenterology, Department of Medicine, Taichung Armed Forces General Hospital, Taichung, Taiwan; 7 School of Nursing, College of Medicine, Chang Gung University, Taoyuan, Taiwan; 8 Department of Pediatrics, Chang Gung Memorial Hospital, Taoyuan, Taiwan; 9 Department of Nursing, Chang Gung Memorial Hospital, Taoyuan, Taiwan; 10 Division of Medical Informatics, Department of Epidemiology, School of Public Health, National Defense Medical Center, Taipei, Taiwan; 11 Big Data Research Center, College of Medicine, Fu-Jen Catholic University, New Taipei City, Taiwan; 12 Department of Public Health, College of Medicine, Fu-Jen Catholic University, New Taipei City, Taiwan; 13 Department of Public Health, China Medical University, Taichung City, Taiwan; 14 Department of Healthcare Administration and Medical Informatics College of Health Sciences, Kaohsiung Medical University, Kaohsiung, Taiwan; 15 Graduate Institute of Clinical Medicine, College of Medicine, Taipei Medical University, Taipei, Taiwan; 16 Cell Physiology and Molecular Image Research Center, Wan Fang Hospital, Taipei Medical University, Taipei, Taiwan; 17 Graduate Institute of Medical Sciences, National Defense Medical Center, Taipei, Taiwan; 18 Graduate Institute of Life Sciences, National Defense Medical Center, Taipei, Taiwan; 19 Center for Biotechnology and Biomedical Engineering, National Central University, Taoyuan, Taiwan; 20 Hualien Armed Forces General Hospital, Xincheng, 97144 Hualien County Taiwan; 21 Division of Colorectal Surgery, Department of Surgery, Tri-Service General Hospital, National Defense Medical Center, Taipei City, Taiwan; 22 School of Medicine, National Defense Medical Center, Taipei City, Taiwan

Correction to: *Scientific Reports* 10.1038/s41598-021-84995-z, published online 31 March 2021

The original version of this Article contained errors in the author list as a previous version of the author list was erroneously published.

The order of author names was incorrect. Chien-An Sun, Chen Lin, and Hung-Che Lin were omitted from the author list. In addition, Yu-Jia Chang was omitted as an equally contributing author. The original author list and affiliations appear below.

Chi-Ming Chu^1,2,3,4,16^, Huan-Ming Hsu^5,6,16^, Chi-Wen Chang^7,8,16^, Yuan-Kuei Li^9,7,16^, Yu-Jia Chang^10,16^, Jyh-Cherng Yu^5^, Chien-Ting Chen^1^, Chen-En Jian^1^, Meng-Chiung Lin^12^, Kang-Hua Chen^7,11^, Ming-Hao Kuo^13^, Chia-Shiang Cheng^14^, Ya-Ting Chang^15^, Yi-Syuan Wu^14^, Hao-Yi Wu^15^, Ya-Ting Yang^15^, Je-Ming Hu^5^ & Yu-Tien Chang^1,2^

^1^Division of Biostatistics and Informatics, Department of Epidemiology, School of Public Health, National Defense Medical Center, No. 161, Sec. 6, Minquan E. Rd., Neihu Dist., Taipei City 11490, Taiwan. ^2^Big Data Research Center, Fu-Jen Catholic University, New Taipei City, Taiwan. ^3^Department of Public Health, China Medical University, Taichung City, Taiwan. ^4^Department of Healthcare Administration and Medical Informatics College of Health Sciences, Kaohsiung Medical University, Kaohsiung, Taiwan. ^5^Division of General Surgery, Department of Surgery, TriService General Hospital, National Defense Medical Center, Taipei, Taiwan. ^6^Department of Surgery, Songshan Branch of Tri-Service General Hospital, National Defense Medical Center, Taipei, Taiwan. ^7^School of Nursing, College of Medicine, Chang Gung University, Taoyuan, Taiwan. ^8^Department of Pediatrics, Chang Gung Memorial Hospital, Taoyuan, Taiwan. ^9^Division of Colorectal Surgery, Department of Surgery, Taoyuan Armed Forces General Hospital, Taoyuan, Taiwan. ^10^Cancer Research Center and Translational Laboratory, Department of Medical Research, Taipei Medical University Hospital, Taipei, Taiwan. ^11^Department of Nursing, Chang Gung Memorial Hospital, TaoYuan, Taiwan. ^12^Division of Gastroenterology, Department of Medicine, Taichung Armed Forces General Hospital, Taichung, Taiwan. ^13^Graduate Institute of Medical Sciences, National Defense Medical Center, Taipei, Taiwan. ^14^Graduate Institute of Life Sciences, National Defense Medical Center, Taipei, Taiwan. ^15^School of Public Health, National Defense Medical Center, Taipei, Taiwan. ^16^These authors contributed equally: Yuan-Kuei Li, Huan-Ming Hsu, Meng-Chiung Lin, Chi-Wen Chang, and Chi-Ming Chu.

The original Article has been corrected.

