# Supplementary material for: Genetic co-expression networks contribute to creating predictive model and exploring novel biomarkers for the prognosis of breast cancer
Source: Sci Rep. 2021 Mar 31;11:7268. doi: 10.1038/s41598-021-84995-z (PMC8012617; doi:10.1038/s41598-021-84995-z)
Supplement: Supplementary file 1 — Supplementary Information. [file 41598_2021_84995_MOESM1_ESM.docx]

# Supplementary Files

# Title

**Genetic co-expression networks contribute to creating predictive model and exploring novel biomarkers for the prognosis of breast cancer**

**Running title: GCN-based modeling exerts greater efficacy of predictions**

Yuan-Kuei Li^a, b*^, nkcell1997@gmail.com

Huan-Ming Hsu^c, d, s*^, hmh0823@gmail.com

Meng-Chiung Lin^e*^, s88.s99@msa.hinet.net

Chi-Wen Chang^f, g, h*^, cwchang0320@gmail.com

Chi-Ming Chu^i, j, k, l, m*^, cm.chu.tw@gmail.com

Yu-Jia Chang^n,o*^, r5424012@tmu.edu.tw

Jyh-Cherng Yu^c^, doc20106@mail.ndmctsgh.edu.tw

Chien-Ting Chen^h^, zxc654951@gmail.com

Chen-En Jian^h^, sm905200@hotmail.com

Chien-An Sun^j^, 040866@mail.fju.edu.tw

Kang-Hua Chen^f, h^, khc@mail.cgu.edu.tw

Ming-Hao Kuo^p^, dadubu3@gmail.com

Chia-Shiang Cheng^q^, bear654328@gmail.com

Ya-Ting Chang^i^, wendy840708@gmail.com

Yi-Syuan Wu^q^, pu1254@gmail.com

Hao-Yi Wu^i^, benny4215@gmail.com

Ya-Ting Yang^i^, yang@mail.ndmctsgh.edu.tw

Chen Lin^b, r^ , clin@ncu.edu.tw

Hung-Che Lin^p, s, t^, lhj50702@gmail.com

Je-Ming Hu^p, s, v^, jeminghu@gmail.com

**Yu-Tien Chang^i, j^, greengarden720925@gmail.com

**Authors’ affiliations:**

^a^Division of Colorectal Surgery, Department of Surgery, Taoyuan Armed Forces General Hospital, Taoyuan, Taiwan

^b^Department of Biomedical Sciences and Engineering, National Central University, Taoyuan, Taiwan

^c^Division of General Surgery, Department of Surgery, Tri-Service General Hospital, National Defense Medical Center, Taipei, Taiwan.

^d^Department of Surgery, Songshan Branch of Tri-Service General Hospital, National Defense Medical Center, Taipei, Taiwan.

^e^Division of Gastroenterology, Department of Medicine, Taichung Armed Forces General Hospital, Taichung, Taiwan.

^f^School of Nursing, College of Medicine, Chang Gung University, Taoyuan, Taiwan

^g^Department of Pediatrics, Chang Gung Memorial Hospital, Taoyuan, Taiwan
^h^Department of Nursing, Chang Gung Memorial Hospital, Tao-Yuan, Taiwan

^i^Division of Medical Informatics, Department of Epidemiology, School of Public Health, National Defense Medical Center, Taipei, Taiwan.

^j^Big Data Research Center, College of Medicine, Fu-Jen Catholic University, New Taipei City, Taiwan
^k^Department of Public Health, College of Medicine, Fu-Jen Catholic University, New Taipei City

^l^Department of Public Health, China Medical University, Taichung City, Taiwan

^m^Department of Healthcare Administration and Medical Informatics College of Health Sciences, Kaohsiung Medical University

^n^Graduate Institute of Clinical Medicine, College of Medicine, Taipei Medical University, Taipei, Taiwan
^o^Cell Physiology and Molecular Image Research Center, Wan Fang Hospital, Taipei Medical University, Taipei, Taiwan

^p^Graduate Institute of Medical Sciences, National Defense Medical Center, Taipei, Taiwan

^q^Graduate Institute of Life Sciences, National Defense Medical Center, Taipei, Taiwan

^r^Center for Biotechnology and Biomedical Engineering, National Central University, Taoyuan, Taiwan

^s^Department of Otolaryngology-Head and Neck Surgery, Tri-Service General Hospital, National Defense Medical Center, Taipei 11490, Taiwan

^t^Hualien Armed Forces General Hospital, Hualien County 97144, Taiwan

^u^Division of Colorectal Surgery, Department of Surgery, Tri-Service General Hospital,  National Defense Medical Center, Taipei City,R.O.C

^v^School of Medicine, National Defense Medical Center, Taipei City, R.O.C

* Equal contribution.

****Corresponding authors:**

Dr. Yu-Tien Chang

Tel.: +886-2-87923126

Address: No.161, Sec. 6, Minquan E. Rd., Neihu Dist., Taipei City 11490, Taiwan (R.O.C.)

Email: greengarden720925@gmail.com

## Table 1: The association of clinical-pathological factors to breast cancer recurrence in multivariable logistic regression.

|  | OR | Sig. |
| --- | --- | --- |
| ER | .728 | .067 |
| Lymph_node | 1.389 | .054 |
| Grade | 1.119 | .291 |
| Size | 1.138 | .006* |
| Age | .996 | .521 |

## Table 2: Forwards conditional multivariable logistic regression on predicting the breast cancer recurrence.

|  | Genes | | |  | Genes + tumor size | | |
| --- | --- | --- | --- | --- | --- | --- | --- |
|  | AUC (%) | ACC (%) | gene # |  | AUC (%) | ACC (%) | gene # |
| Model 1 | 74.9 | 70.3 | 9 |  | 62.6 | 66.9 | 1 |
| Model 2 | 80.4 | 73.5 | 10 |  | 70.8 | 70.9 | 10 |
| Model 3 | 84 | 75.6 | 32 |  | 75.7 | 72.2 | 11 |
| Model 4 | 86 | 77.9 | 43 |  | 78.6 | 73.7 | 14 |

AUC: area under curve of ROC curve

ACC: accuracy

gene # : the number of genes included in the model

The gene pool of creating Model 1 to 4 are from the GCNs of 34 key candidate genes, r>0.82, r>0.80 and r>0.79 respectively.

## Table 3: Cox regression of the risk scores of each model.

| Cox regression | Model 1 | | Model 2 | | Model 3 | | Model 4 | | Model 5 | | Model 6 | |
| --- | --- | --- | --- | --- | --- | --- | --- | --- | --- | --- | --- | --- |
|  | **Risk Score** | | | | | | | | | | | |
|  | Cont. | High Risk | Cont. | High Risk | Cont. | High Risk | Cont. | High Risk | Cont. | High Risk | Cont. | High Risk |
| B* | 0.98 | 0.64 | 1.06 | 0.72 | 1.07 | 0.88 | 1.18 | 1.2 | 0.99 | 0.62 | 1.03 | 0.72 |
| HR* | 2.66 | 1.89 | 2.89 | 2.05 | 2.92 | 2.41 | 3.25 | 3.32 | 2.69 | 1.85 | 2.8 | 2.06 |
| se(B)* | 0.16 | 0.11 | 0.12 | 0.11 | 0.1 | 0.11 | 0.09 | 0.12 | 0.12 | 0.11 | 0.14 | 0.11 |
| P value* | 2.26E-09 | 6.05E-09 | 0 | 8.24E-11 | 0 | 5.00E-15 | 0 | 0 | 0 | 1.16E-08 | 3.77E-14 | 8.37E-11 |
| B | 0.99 | 0.65 | 1.07 | 0.72 | 1.05 | 0.87 | 1.18 | 1.19 | 0.98 | 0.62 | 1.01 | 0.71 |
| HR | 2.69 | 1.91 | 2.92 | 2.06 | 2.86 | 2.39 | 3.26 | 3.28 | 2.66 | 1.86 | 2.76 | 2.03 |
| se(B) | 0.16 | 0.11 | 0.12 | 0.11 | 0.1 | 0.11 | 0.09 | 0.12 | 0.12 | 0.11 | 0.14 | 0.11 |
| P value | 1.34E-09 | 2.78E-09 | 0 | 5.47E-11 | 0 | 6.66E-15 | 0 | 0 | 0 | 8.13E-09 | 6.12E-14 | 2.09E-10 |

*Under the control of the node status.

HR: harzard ratio of Cox proportional hazards model; p: p value of Cox proportional hazards model; B: coefficients of Cox proportional hazards model.

Cont.: risk scores in continuous variables. The continuous risk group was categorized into high and low risk groups using a cutoff point of zero.

## Table 4: The GCN-based prediction models on breast cancer recurrences using Cox proportional hazards model and various size network of genetic predictors.

| Genes | Model 1  34 KCGs | | | Model 2  r > 0.82 | | | Model 3  r >0.80 | | | Model 4  r >0.79 | | | Model 5^#^ | | |
| --- | --- | --- | --- | --- | --- | --- | --- | --- | --- | --- | --- | --- | --- | --- | --- |
|  | B | HR | p | B | HR | p | B | HR | p | B | HR | p | B | HR | p |
| Node | 0.49 | 1.64 | 0.005 | 0.41 | 1.5 | 0.02 | 0.59 | 1.81 | 9.22E-04 | 0.54 | 1.72 | 0.004 | 0.46 | 1.59 | 0.008 |
| ***AACS*** | 0.4 | 1.49 | 0.03 | 0.47 | 1.59 | 0.01 | 0.64 | 1.89 | 7.59E-04 | 0.55 | 1.73 | 0.01 | 0.46 | 1.58 | 0.01 |
| ***ABCC1*** |  |  |  |  |  |  | 0.39 | 1.47 | 0.04 |  |  |  |  |  |  |
| *BARD1* |  |  |  |  |  |  |  |  |  | 0.4 | 1.49 | 0.01 |  |  |  |
| ***C10orf3*** | -0.6 | 0.55 | 0.001 | -0.78 | 0.46 | 1.22E-04 | -0.73 | 0.48 | 4.27E-04 | -0.87 | 0.42 | 5.18E-05 | -0.47 | 0.62 | 0.005 |
| *C10orf56** |  |  |  |  |  |  | 0.83 | 2.28 | 3.63E-04 |  |  |  |  |  |  |
| *C8orf32* |  |  |  |  |  |  |  |  |  | -0.49 | 0.61 | 0.01 |  |  |  |
| *C8orf33* |  |  |  |  |  |  |  |  |  | 0.42 | 1.53 | 0.005 |  |  |  |
| *CCNA2** |  |  |  | 0.67 | 1.95 | 0.002 | 0.57 | 1.76 | 0.01 |  |  |  |  |  |  |
| *CCNB1* |  |  |  | 0.4 | 1.49 | 0.02 | 0.46 | 1.58 | 0.01 | 0.52 | 1.68 | 0.01 |  |  |  |
| *CCNE2** |  |  |  |  |  |  | 0.47 | 1.61 | 0.02 | 0.84 | 2.33 | 1.14E-04 | 0.47 | 1.60 | 0.008 |
| *CCT6A* |  |  |  |  |  |  |  |  |  | 0.62 | 1.86 | 0.04 |  |  |  |
| *CEBPZ* |  |  |  |  |  |  |  |  |  | -0.4 | 0.67 | 0.02 |  |  |  |
| *CHEK1* |  |  |  | -0.31 | 0.73 | 0.04 |  |  |  |  |  |  |  |  |  |
| *COX4NB* |  |  |  |  |  |  |  |  |  | 0.61 | 1.84 | 0.003 |  |  |  |
| *CPZ* |  |  |  | 0.37 | 1.45 | 0.01 |  |  |  |  |  |  |  |  |  |
| *CXCL10* |  |  |  |  |  |  |  |  |  | 0.4 | 1.5 | 9.97E-04 |  |  |  |
| ***DEAF1*** | -0.29 | 0.75 | 0.02 | -0.35 | 0.71 | 0.02 | -0.34 | 0.71 | 0.02 | -0.61 | 0.54 | 9.17E-05 |  |  |  |
| *DTX3* |  |  |  |  |  |  |  |  |  | -0.62 | 0.54 | 0.004 |  |  |  |
| *EBP** |  |  |  |  |  |  |  |  |  | -1.31 | 0.27 | 7.07E-06 |  |  |  |
| ***EEF1E1*** |  |  |  | 0.79 | 2.21 | 1.36E-04 | 0.85 | 2.34 | 3.44E-05 | 0.73 | 2.07 | 0.004 | 0.41 | 1.50 | 0.003 |
| *EIF4E2* |  |  |  |  |  |  |  |  |  | -0.77 | 0.46 | 0.002 |  |  |  |
| *FCER1G* |  |  |  |  |  |  | 0.61 | 1.84 | 0.003 |  |  |  |  |  |  |
| *FLJ20641* |  |  |  |  |  |  |  |  |  | -0.64 | 0.53 | 0.002 |  |  |  |
| *GGCX* |  |  |  |  |  |  |  |  |  | 0.66 | 1.93 | 0.004 |  |  |  |
| *GGTLA1* |  |  |  |  |  |  |  |  |  | 0.39 | 1.47 | 0.003 |  |  |  |
| *IDUA** |  |  |  |  |  |  | 0.64 | 1.9 | 7.22E-06 | 0.44 | 1.55 | 0.01 | 0.67 | 1.96 | 5.34E-07 |
| ***IGKC*** |  |  |  | -0.21 | 0.81 | 0.01 |  |  |  |  |  |  |  |  |  |
| ***KIAA0101*** | 0.39 | 1.47 | 8.05E-05 | 0.43 | 1.54 | 0.01 | 0.35 | 1.42 | 0.05 |  |  |  |  |  |  |
| ***KIF11*** | 0.54 | 1.72 | 0.003 |  |  |  |  |  |  | 0.53 | 1.69 | 0.04 |  |  |  |
| *KIF14** |  |  |  |  |  |  | -0.51 | 0.6 | 0.01 | -0.7 | 0.5 | 8.32E-04 |  |  |  |
| *LILRB3* |  |  |  |  |  |  |  |  |  | -0.47 | 0.62 | 0.01 |  |  |  |
| ***LMNB1*** | 0.46 | 1.58 | 0.01 | 0.61 | 1.84 | 0.002 | 0.5 | 1.64 | 0.02 | 0.73 | 2.07 | 6.67E-04 | 0.67 | 1.95 | 8.36E-04 |
| *MGC26885* |  |  |  |  |  |  | 0.54 | 1.71 | 0.03 |  |  |  |  |  |  |
| *MGC27165** |  |  |  |  |  |  | -0.74 | 0.48 | 1.79E-04 | -0.79 | 0.45 | 1.81E-04 | -0.69 | 0.50 | 1.25E-06 |
| *NCOA6IP* |  |  |  |  |  |  |  |  |  | -0.61 | 0.54 | 0.003 |  |  |  |
| *NUP153* |  |  |  |  |  |  |  |  |  | -0.42 | 0.65 | 0.04 |  |  |  |
| *PCNA* |  |  |  |  |  |  | 0.41 | 1.51 | 0.02 |  |  |  |  |  |  |
| *RFC2* |  |  |  | 0.53 | 1.7 | 0.02 |  |  |  |  |  |  |  |  |  |
| *RORC** |  |  |  |  |  |  |  |  |  | -0.83 | 0.44 | 1.09E-05 | -0.68 | 0.51 | 5.36E-05 |
| *TIMELESS* |  |  |  | -0.61 | 0.54 | 0.02 |  |  |  |  |  |  |  |  |  |
| *TPCN1* |  |  |  |  |  |  |  |  |  | 0.66 | 1.93 | 0.003 |  |  |  |
| *TRBV21_1* |  |  |  |  |  |  |  |  |  | -0.33 | 0.72 | 8.48E-04 |  |  |  |
| *TTC10* |  |  |  |  |  |  |  |  |  | -0.62 | 0.54 | 0.005 |  |  |  |
| *UCHL5* |  |  |  |  |  |  |  |  |  | 0.54 | 1.71 | 0.04 |  |  |  |
| *VPS41* |  |  |  |  |  |  |  |  |  | -0.61 | 0.54 | 0.03 |  |  |  |
| *ZMYND11* |  |  |  |  |  |  |  |  |  | -0.72 | 0.49 | 0.01 |  |  |  |
| R square | 0.05 |  |  | 0.09 |  |  | 0.12 |  |  | 0.21 |  |  | 0.08 |  |  |
| Likelihood ratio | 44.55 |  |  | 81.98 |  |  | 116.8 |  |  | 209 |  |  | 73.77 |  |  |
| df | 7 |  |  | 14 |  |  | 18 |  |  | 35 |  |  | 9 |  |  |
| p of Likelihood ratio test | <0.001 |  |  | 1.21E-11 |  |  | 2.22E-16 |  |  | ~0 |  |  | 2.77E-12 |  |  |

34 candidate genes are marked in bold; HR: harzard ratio of Cox proportional hazards model; p: p value of Cox proportional hazards model; B: coefficients of Cox proportional hazards model.

^#^Final model: the genes were selected from 46 genes from model 1-4 and the criteria were set as VIF<10, the chosen significance level for entry (SLE) < 0.08 and the chosen significance level for stay (SLS) < 0.05 using the My.stepwise.coxph function of the My.stepwise package for R statistical software.

^*^ Significant genes of interests highly co-expressed with 34 candidate genes on predicting BC recurrenc

## Table 5: Relative importance of the significant genes in models 1 to 4.

| **Significant genes** | **Model 1** | **Model 2** | **Model 3** | **Model 4** | **Sum of importance** | **Average of importance** |
| --- | --- | --- | --- | --- | --- | --- |
| ***KIAA0101*** |  | 0.034 | 0.078 | 0.339 | 0.452 | 0.151 |
| ***C10orf3*** | 0.082 | 0.105 | 0.178 | 0.227 | 0.592 | 0.148 |
| ***EEF1E1*** | 0.041 | 0.145 | 0.175 |  | 0.362 | 0.121 |
| *C10orf56* |  | 0.108 |  |  | 0.108 | 0.108 |
| ***KIF11*** | 0.021 |  |  | 0.186 | 0.208 | 0.104 |
| *IDUA* | 0.033 | 0.171 |  |  | 0.203 | 0.102 |
| *EBP* | 0.101 |  |  |  | 0.101 | 0.101 |
| *RORC* | 0.097 |  |  |  | 0.097 | 0.097 |
| *MGC27165* | 0.070 | 0.119 |  |  | 0.189 | 0.095 |
| *Node_F* | 0.042 | 0.093 | 0.064 | 0.176 | 0.375 | 0.094 |
| ***LMNB1*** | 0.058 | 0.050 | 0.111 | 0.134 | 0.352 | 0.088 |
| *CPZ* |  |  | 0.086 |  | 0.086 | 0.086 |
| *CCNA2* |  | 0.056 | 0.115 |  | 0.171 | 0.085 |
| ***AACS*** | 0.037 | 0.096 | 0.076 | 0.105 | 0.314 | 0.078 |
| ***DEAF1*** | 0.077 | 0.047 | 0.069 | 0.115 | 0.308 | 0.077 |
| ***IGKC*** |  |  | 0.074 |  | 0.074 | 0.074 |
| *FCER1G* |  | 0.073 |  |  | 0.073 | 0.073 |
| *TIMELESS* |  |  | 0.065 |  | 0.065 | 0.065 |
| *RFC2* |  |  | 0.065 |  | 0.065 | 0.065 |
| *CCNE2* | 0.075 | 0.049 |  |  | 0.124 | 0.062 |
| *KIF14* | 0.056 | 0.060 |  |  | 0.116 | 0.058 |
| *TRBV21_1* | 0.056 |  |  |  | 0.056 | 0.056 |
| *CXCL10* | 0.054 |  |  |  | 0.054 | 0.054 |
| *CCNB1* | 0.039 | 0.059 | 0.062 |  | 0.160 | 0.053 |
| *EIF4E2* | 0.050 |  |  |  | 0.050 | 0.050 |
| *CHEK1* |  |  | 0.050 |  | 0.050 | 0.050 |
| *FLJ20641* | 0.049 |  |  |  | 0.049 | 0.049 |
| *PCNA* |  | 0.045 |  |  | 0.045 | 0.045 |
| *GGTLA1* | 0.044 |  |  |  | 0.044 | 0.044 |
| *COX4NB* | 0.044 |  |  |  | 0.044 | 0.044 |
| *TPCN1* | 0.044 |  |  |  | 0.044 | 0.044 |
| *GGCX* | 0.042 |  |  |  | 0.042 | 0.042 |
| *NCOA6IP* | 0.042 |  |  |  | 0.042 | 0.042 |
| *MGC26885* |  | 0.042 |  |  | 0.042 | 0.042 |
| *DTX3* | 0.042 |  |  |  | 0.042 | 0.042 |
| *C8orf33* | 0.040 |  |  |  | 0.040 | 0.040 |
| *TTC10* | 0.040 |  |  |  | 0.040 | 0.040 |
| *BARD1* | 0.038 |  |  |  | 0.038 | 0.038 |
| *ZMYND11* | 0.038 |  |  |  | 0.038 | 0.038 |
| ***ABCC1*** |  | 0.037 |  |  | 0.037 | 0.037 |
| *LILRB3* | 0.035 |  |  |  | 0.035 | 0.035 |
| *C8orf32* | 0.032 |  |  |  | 0.032 | 0.032 |
| *CEBPZ* | 0.026 |  |  |  | 0.026 | 0.026 |
| *VPS41* | 0.022 |  |  |  | 0.022 | 0.022 |
| *NUP153* | 0.022 |  |  |  | 0.022 | 0.022 |
| *CCT6A* | 0.021 |  |  |  | 0.021 | 0.021 |
| *UCHL5* | 0.020 |  |  |  | 0.020 | 0.020 |

The genes are listed with the value of the average of importance in descending order. The significant genes from the 34 KCGs are marked in bold. The top 12 important hub genes for BC recurrence are marked in red.

## Table 6: Description of the integrated GSE data sets.

| Variables | | Data sets, n(%) | | | | | | | |
| --- | --- | --- | --- | --- | --- | --- | --- | --- | --- |
|  |  | Wang et al.[[18](#_ENREF_18)]  GSE2034 | | Sotiriou et al.[[20](#_ENREF_20)]  GSE2990 | | Ivshina et al. [[21](#_ENREF_21)]  GSE4922 | | Desmedt et al. [[19](#_ENREF_19)]  GSE7390 | |
| **Recurrence at the end of follow-up^#^** | 0 | 179 | 62.6 | 120 | 64.2 | 160 | 64.3 | 107 | 54.0 |
|  | 1 | 107 | 37.4 | 67 | 35.8 | 89 | 35.7 | 91 | 46.0 |
| **Node** | Negative | 286 | 100.0 | 153 | 83.6 | 159 | 66.3 | 198 | 100.0 |
|  | Positive | 0 | 0 | 30 | 16.4 | 81 | 33.8 | 0 | 0 |
| **Follow-up* time,** mean(sd) |  | 6.46(3.52) | | 6.62(3.95) | | 7.14(4.30) | | 9.31(5.56) | |

^#^Chi-square: tests the difference between recurrence status and the datasets, p=0.104。

^*^ANOVA: tests the follow-up time difference between the datasets, p<0.001

## Table 7: Description of the origin of the 34 candidate genes

| **Author** | **Study** | **Proportion of overlapped genes** | **Overlapped genes** |
| --- | --- | --- | --- |
| **Chou et al. [**[**14**](#_ENREF_14)**]** | Gene expression profiling of breast cancer survivability by pooled cDNA microarray analysis using logistic regression, artificial neural networks and decision trees (our previous study) | 21/21 | LMCD1, DEAF1, AP2A2, LMNB1, ZFP36L2, ABCC1, PLOD2, LARS2, CDCA3, AACS, TNFRSF25, SMC1A, ADIPOQ, DPP3, FADD, PLK1, SDS, HSPB6, MTERFD1, CHPF, AQP1. |
| **Wang et al.[**[**18**](#_ENREF_18)**]**  **Desmedt et al. [**[**19**](#_ENREF_19)**]** | Gene-expression profiles to predict distant metastasis of lymph-node-negative primary breast cancer    Strong time dependence of the 76-gene prognostic signature for node-negative breast cancer patients in the TRANSBIG multicenter independent validation series | 5/76 | AP2A2, LST1, MLF1IP, PLK1, ZFP36L2 |
| **Sotiriou et al. [**[**20**](#_ENREF_20)**]** | Gene expression profiling in breast cancer: understanding the molecular basis of histologic grade to improve prognosis | 19/97 | ASPM, BUB1B, C10orf3, CCNB1, CDCA3, CDKN3, HMMR, KIF11, KIF20A, KIF4A, LMNB1, MAD2L1, MELK, MLF1IP, PLK1, PRC1, PSF1, RACGAP1, STK6, |
| **Ivshina et al. [**[**21**](#_ENREF_21)**]** | Genetic reclassification of histologic grade delineates new clinical subtypes of breast cancer | 20/232 | ASPM, BUB1B, C10orf3, CCNB1, CDCA3, CDKN3, CNIH4, HMMR, KIAA0101, KIF11, KIF20A, KIF4A, LMNB1, MAD2L1, MELK, PRC1, PSF1, RACGAP1, STK6, TOP2A |
| **Xu et al. [**[**22**](#_ENREF_22)**]** | Merging microarray data from separate breast cancer studies provides a robust prognostic test | 13/112 | AP2A2,EEF1E1,IGHM,IGKC,LST1,MLF1IP,RACGAP1,STK6,CDKN3,ASPM,MAD2L1,MELK,PRC1 |

## Table 8: Top 100 significant genes related to BC recurrence in the Chou et al. study [[14](#_ENREF_14)].

| No. | Gene Symbol | GenBank ID | Annotation |
| --- | --- | --- | --- |
| 1 | ***AACS*** | NM_023928 | acetoacetyl-CoA synthetase |
| 2 | ***ABCC1*** | AI539710 | ATP-binding cassette, sub-family C (CFTR/MRP), member 1 |
| 6 | ***AP2A2*** | BC006155 | adaptor-related protein complex 2, alpha 2 subunit |
| 9 | ***ASPM*** | NM_018123 | asp (abnormal spindle)-like, microcephaly associated (Drosophila) |
| 14 | ***BUB1B*** | NM_001211 | BUB1 budding uninhibited by benzimidazoles 1 homolog beta (yeast) |
| 19 | ***CCNB1*** | BE407516 | cyclin B1 |
| 20 | ***CDCA3*** | NM_031299 | cell division cycle associated 3 |
| 21 | ***CDKN3*** | AF213033 | cyclin-dependent kinase inhibitor 3 (CDK2-associated dual specificity phosphatase) |
| 23 | ***CNIH4*** | NM_014184 | cornichon homolog 4 (Drosophila) |
| 26 | ***DEAF1*** | AF068892 | deformed epidermal autoregulatory factor 1 (Drosophila) |
| 30 | ***EEF1E1*** | NM_004280 | eukaryotic translation elongation factor 1 epsilon 1 |
| 41 | ***HMMR*** | NM_012485 | hyaluronan-mediated motility receptor (RHAMM) |
| 45 | ***IGHM*** | BC001872 | immunoglobulin heavy constant mu |
| 46 | ***IGKC*** | AF103574 | Immunoglobulin kappa variable 1-5 |
| 48 | ***KIAA0101*** | NM_014736 | KIAA0101 |
| 49 | ***KIF11*** | NM_004523 | kinesin family member 11 |
| 50 | ***KIF20A*** | NM_005733 | kinesin family member 20A |
| 51 | ***KIF4A*** | NM_012310 | kinesin family member 4A |
| 53 | ***LARS2*** | NM_015340 | leucyl-tRNA synthetase 2, mitochondrial |
| 54 | ***LMCD1*** | NM_014583 | LIM and cysteine-rich domains 1 |
| 55 | ***LMNB1*** | NM_005573 | lamin B1 |
| 57 | ***LST1*** | AF000425 | leukocyte specific transcript 1 |
| 59 | ***MAD2L1*** | NM_002358 | MAD2 mitotic arrest deficient-like 1 (yeast) |
| 61 | ***MELK*** | NM_014791 | maternal embryonic leucine zipper kinase |
| 69 | ***PLK1*** | NM_005030 | polo-like kinase 1 (Drosophila) |
| 70 | ***PLOD2*** | AI754404 | procollagen-lysine, 2-oxoglutarate 5-dioxygenase 2 |
| 73 | ***PRC1*** | NM_003981 | protein regulator of cytokinesis 1 |
| 76 | ***RACGAP1*** | AU153848 | Rac GTPase activating protein 1 |
| 94 | ***TOP2A*** | AU159942 | topoisomerase (DNA) II alpha 170 kDa |
| 98 | ***ZFP36L2*** | AI356398 | zinc finger protein 36, C3H type-like 2 |
| 3 | *ADIPOQ* | NM_004797 | adiponectin, C1Q and collagen domain containing |
| 4 | *ADM* | NM_001124 | adrenomedullin |
| 5 | *AGPAT2* | U56418 | 1-acylglycerol-3-phosphate O-acyltransferase 2 (lysophosphatidic acid acyltransferase, beta) |
| 7 | *AQP1* | NM_000385 | aquaporin 1 (channel-forming integral protein, 28 kDa) |
| 8 | *ARMC8* | BF195973 | armadillo repeat containing 8 |
| 10 | *AXL* | AI467916 | AXL receptor tyrosine kinase |
| 11 | *BCAN* | NM_021948 | brevican |
| 12 | *BTBD3* | NM_014962 | BTB (POZ) domain containing 3 |
| 13 | *BTG2* | BG339064 | BTG family, member 2 |
| 15 | ***C10orf3*** | NM_018131 | chromosome 10 open reading frame 3 |
| 16 | *C6orf142* | AJ408433 | chromosome 6 open reading frame 142 |
| 17 | *CACYBP* | AF057356 | calcyclin binding protein |
| 18 | *CCL21* | NM_002989 | chemokine (C-C motif) ligand 21 |
| 22 | *CHPF* | NM_024536 | chondroitin polymerizing factor |
| 24 | *CPOX* | NM_000097 | coproporphyrinogen oxidase |
| 25 | *CPSF6* | AU149367 | cleavage and polyadenylation specific factor 6, 68 kDa |
| 27 | *DPP3* | NM_005700 | dipeptidylpeptidase 3 |
| 28 | *DPY19L4* | AI669947 | dpy-19-like 4 (C. elegans) |
| 29 | *DTX3* | N92708 | deltex 3 homolog (Drosophila) |
| 31 | *EIF2C2* | AI613483 | Eukaryotic translation initiation factor 2C, 2 |
| 32 | *EIF4E* | AW268640 | eukaryotic translation initiation factor 4E |
| 33 | *FADD* | NM_003824 | Fas (TNFRSF6)-associated via death domain |
| 34 | *FBLN5* | NM_006329 | fibulin 5 |
| 35 | *GLRX2* | NM_016066 | glutaredoxin 2 |
| 36 | *GMNN* | NM_015895 | geminin, DNA replication inhibitor |
| 37 | *GPR27* | NM_018971 | G protein-coupled receptor 27 |
| 38 | *GPSM2* | NM_013296 | G-protein signalling modulator 2 (AGS3-like, C. elegans) |
| 39 | *HIPK2* | R37104 | Homeodomain interacting protein kinase 2 |
| 40 | *HLA-DQB1* | M16276 | Major histocompatibility complex, class II, DQ beta 1 |
| 42 | *HSPB6* | AL551046 | heat shock protein, alpha-crystallin-related, B6 |
| 43 | *IGHA1* | AF343666 | immunoglobulin heavy constant alpha 1 |
| 44 | *IGHG3* | M87789 | Immunoglobulin heavy constant gamma 3 (G3m marker) |
| 47 | *IGLC2* | D87023 | Immunoglobulin lambda joining 2 |
| 52 | *LARP4* | AL050205 | La ribonucleoprotein domain family, member 4 |
| 56 | *LOC391427* | XM_372952 | similar to Ig kappa chain precursor V region (orphon V108) - human (fragment) |
| 58 | *LYL1* | BC002796 | lymphoblastic leukemia derived sequence 1 |
| 60 | *ME1* | AL049699 | Malic enzyme 1, NADP(+)-dependent, cytosolic |
| 62 | *MGC39900* | BF677486 | hypothetical protein MGC39900 |
| 63 | ***MLF1IP*** | NM_024629 | MLF1 interacting protein |
| 64 | *MRPS17* | NM_015969 | mitochondrial ribosomal protein S17 |
| 65 | *MTERFD1* | NM_015942 | MTERF domain containing 1 |
| 66 | *NETO2* | NM_018092 | neuropilin (NRP) and tolloid (TLL)-like 2 |
| 67 | *PARG* | NM_003631 | poly (ADP-ribose) glycohydrolase |
| 68 | *PARP3* | AF083068 | poly (ADP-ribose) polymerase family, member 3 |
| 71 | *PMM2* | NM_000303 | phosphomannomutase 2 |
| 72 | *POLD3* | D26018 | polymerase (DNA-directed), delta 3, accessory subunit |
| 74 | ***PSF1*** | NM_021067 | DNA replication complex GINS protein PSF1 |
| 75 | *PTPRN2* | NM_002847 | protein tyrosine phosphatase, receptor type, N polypeptide 2 |
| 77 | *RAD54B* | NM_012415 | RAD54 homolog B (S. cerevisiae) |
| 78 | *RCN2* | BC004892 | reticulocalbin 2, EF-hand calcium binding domain |
| 79 | *RFC4* | NM_002916 | replication factor C (activator 1) 4, 37 kDa |
| 80 | *RORC* | NM_005060 | RAR-related orphan receptor C |
| 81 | *SDS* | NM_006843 | serine dehydratase |
| 82 | *SEC24A* | AJ131244 | SEC24 related gene family, member A (S. cerevisiae) |
| 83 | *SIPA1* | NM_006747 | signal-induced proliferation-associated gene 1 |
| 84 | *SLC25A32* | NM_030780 | solute carrier family 25, member 32 |
| 85 | *SLIT2* | AF055585 | slit homolog 2 (Drosophila) |
| 86 | *SMC1A* | NM_006306 | structural maintenance of chromosomes 1A |
| 87 | *STK3* | NM_006281 | serine/threonine kinase 3 (STE20 homolog, yeast) |
| 88 | ***STK6*** | NM_003600 | serine/threonine kinase 6 |
| 89 | *TBK1* | NM_013254 | TANK-binding kinase 1 |
| 90 | *TDG* | NM_003211 | thymine-DNA glycosylase |
| 91 | *TMEM70* | BC002748 | transmembrane protein 70 |
| 92 | *TMEM93* | NM_031298 | transmembrane protein 93 |
| 93 | *TNFRSF25* | U94506 | tumor necrosis factor receptor superfamily, member 25 |
| 95 | *TPSAB1* | NM_003294 | tryptase alpha/beta 1 |
| 96 | *VPS13B* | AI052003 | vacuolar protein sorting 13B (yeast) |
| 97 | *VRK2* | NM_006296 | vaccinia related kinase 2 |
| 99 | *ZNF137* | NM_003438 | zinc finger protein 137 (clone pHZ-30) |
| 100 | *ZNF468* | BE541042 | zinc finger protein ZNF468 |

34 candidate genes are in bold.


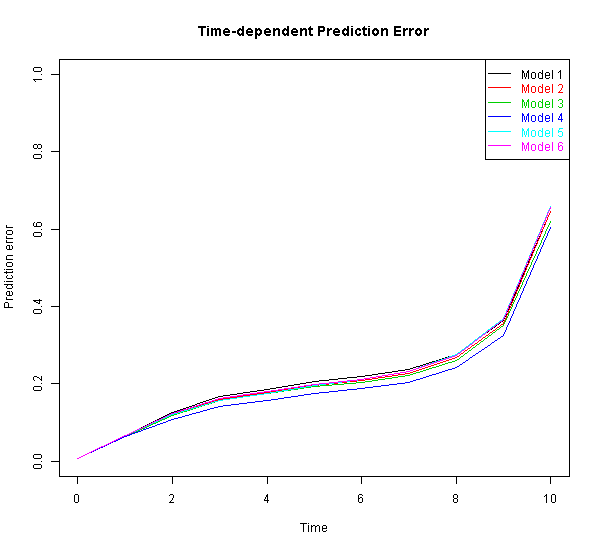


## Figure 1 **Time-dependent Prediction errors of the risk scores.** Model 6 refers to the model from Chou et al. with a 21-gene signature.


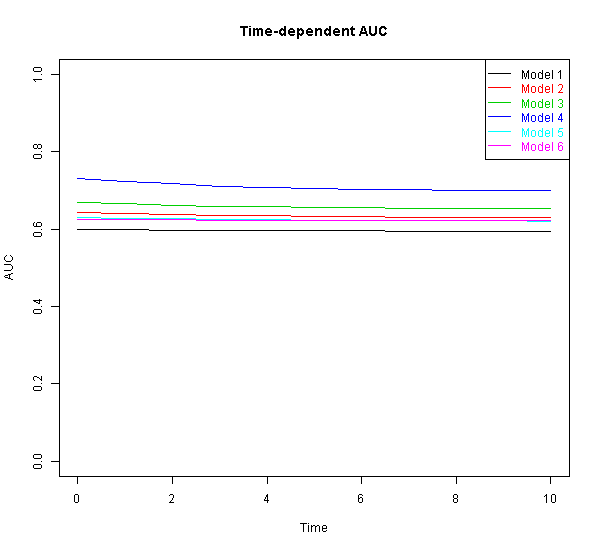


## Figure 2 **Time-dependent AUC of the risk scores.** Model 6 refers to the model from Chou’s study [[14](#_ENREF_14)] using 21-gene signature.


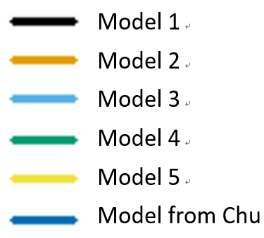

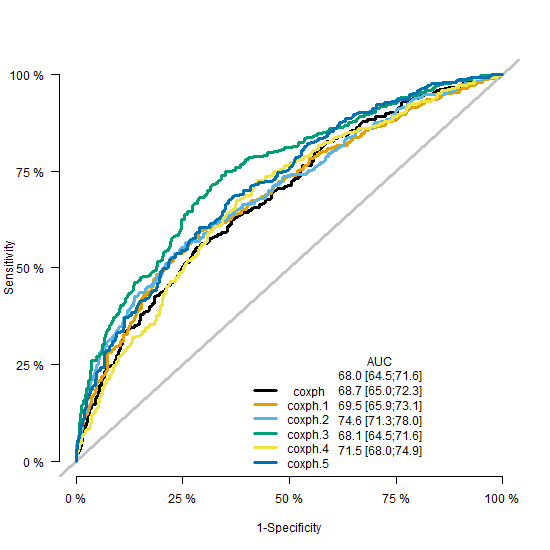


3-year RFS


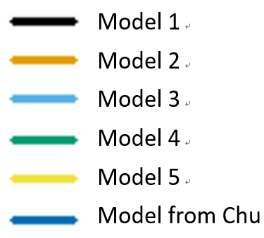

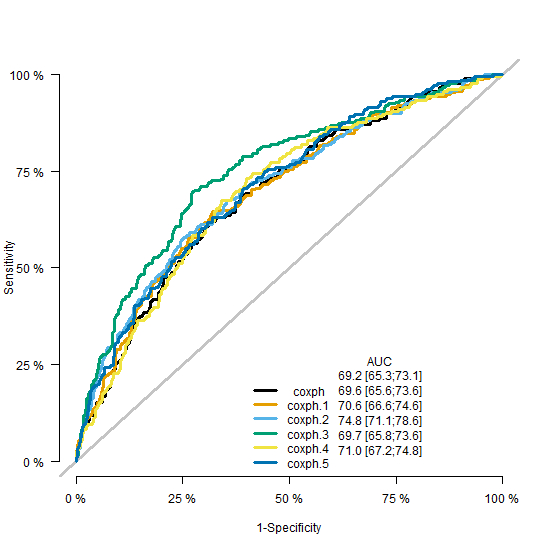
5-year RFS


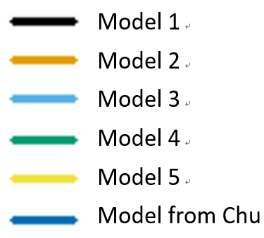

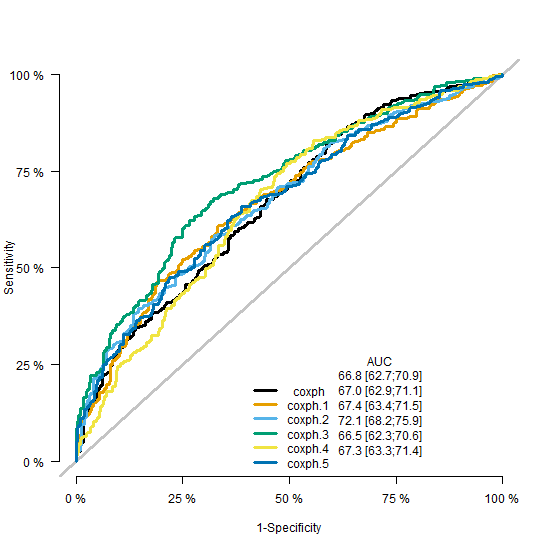


10-year RFS

## Figure 3 The ROC plots of model 1-5 and model Chou’s study on predicting 3,5,10 year RFS in BC.


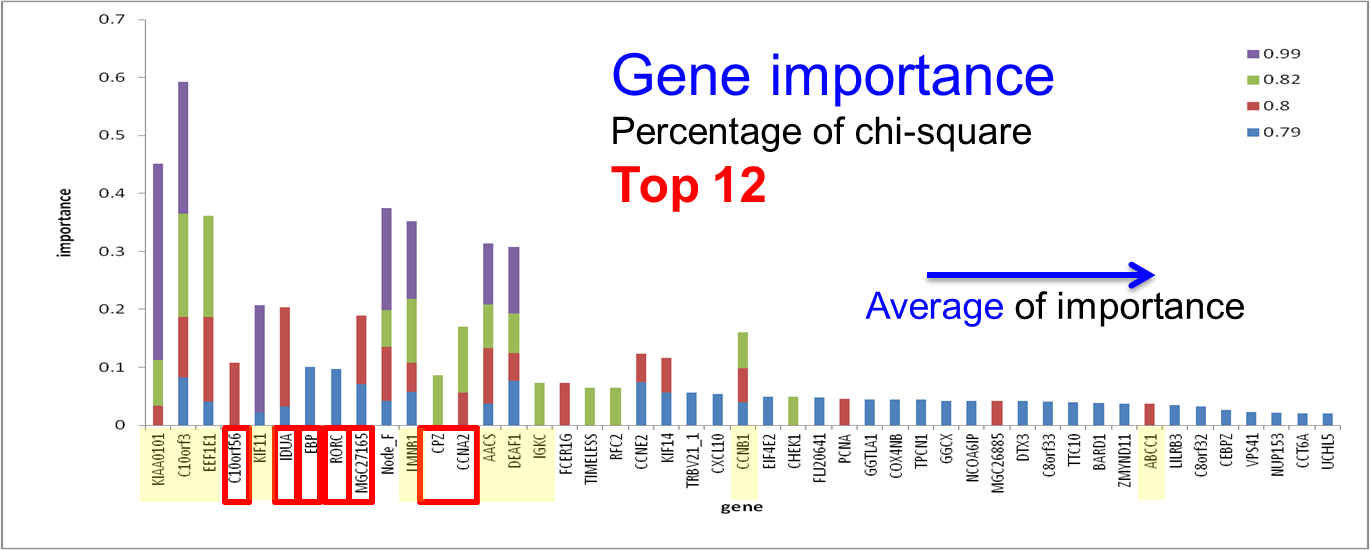


(a)


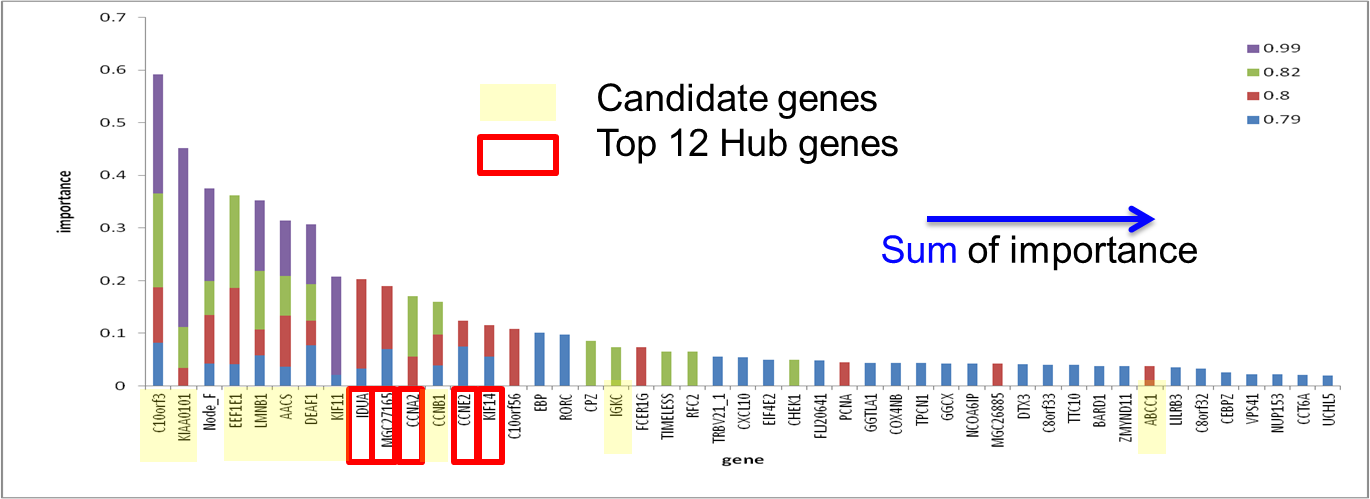


(b)

## Figure 4: Relative importance of genes. (a) The genes are listed with the value of average importance of a gene in descending order. (b) The genes are listed with the value of the sum of the importance of a gene in descending order. Genes belonging to 34 KCGs are in yellow blocks, and the top 12 hub genes are in red rectangles.
